# CircRNA LDLR promotes proliferation and aerobic glycolysis of gastric cancer cells by targeting CHD1 with miR-449b-5p

**DOI:** 10.55730/1300-0152.2681

**Published:** 2023-12-07

**Authors:** FanYe ZENG, JunTao ZHAO, MengTing TONG, WenTing HE, Nan LI, YuXiang FAN, YanHua ZHU, LiPing ZHANG, HongLiang ZHANG

**Affiliations:** Department of Oncology, Affiliated Hospital of Traditional Chinese Medicine of Xinjiang Medical University, Urumqi City, Xinjiang Uygur Autonomous Region, China

**Keywords:** CircRNA, LDLR, aerobic glycolysis, miR-449b-5p, CHD1

## Abstract

**Background/aim:**

Circular RNAs can serve as detection biomarkers and therapeutic targets for tumors. Our study aimed to elucidate the mechanisms associated with circRNA LDLR (circLDLR) in gastric cancer (GC) proliferation and aerobic glycolysis.

**Materials and methods:**

Expression signatures of circLDLR, miR-449b-5p, and CHD1 were examined in GC samples using quantitative PCR. Proliferation ability of MKN-45 cells was assessed via CCK-8 and EdU assays, and cell apoptosis was measured by flow cytometry. Glucose uptake, lactate production, ATP/ADP ratios, and NAD^+^/NADH ratios in cell supernatants were quantified to evaluate aerobic glycolysis. Subcellular isolation assay, quantitative PCR, immunoblot analysis, RNA immunoprecipitation (RIP), and dual luciferase reporter assay were employed to investigate the relationship between genes.

**Results:**

Expression of circLDLR and CHD1 was elevated, while miR-449b-5p expression decreased in GC. Functionally, overexpression of circLDLR enhanced proliferation and aerobic glycolysis and hampered apoptosis of MKN-45 cells. However, upregulation of miR-449b-5p or downregulation of CHD1 reversed these effects. CircLDLR acted as an miRNA spongeand regulated the expression of miR-449b-5p, thereby affecting CHD1 and accelerating GC malignant progression.

**Conclusion:**

CircLDLR drives the proliferation and aerobic glycolysis of GC cells by targeting CHD1 with miR-449b-5p, which is an ideal potential target for early diagnosis and clinical treatment of GC.

## 1. Introduction

According to a 2020 CANCER TODAY report (https://gco.iarc.fr/today), gastric cancer (GC) ranks fifth in terms of prevalence and fourth in terms of death rate globally. Since advanced cases of GC are primarily diagnosed, there is a critical need for diagnostic biomarkers that are highly efficient and reliable to improve prognosis and the 5-year survival rate (Liu et al., 2010; Zhang et al., 2020). Under aerobic conditions, glucose is often converted into lactic acid, a process known as the Warburg effect, which is the main metabolic mode to favor tumor cell survival (Vasudevan et al., 2007; Vander Heiden et al., 2009; Liu et al., 2018). It is generally believed that Warburg effect is related to poor prognosis and chemotherapy resistance in GC (Zeng et al., 2018). However, the precise pathogenesis of GC and the specific mechanism of action between GC and Warburg effect remain unclear. Therefore, exploring the molecular mechanisms of GC is advantageous for a niche to develop biomarkers for early diagnosis and potential therapeutic targets.

circRNA is a single-stranded closed-loop RNA mainly located in the cytoplasm, with high abundance, stability, and unique expression characteristics related to cancer progression and prognosis (Hansen et al., 2013; Skene et al., 2014; Hsiao et al., 2017). circRNA acts as ceRNA to inhibit miRNA and regulate gene expression at the posttranscriptional level (Cheng et al., 2019; Jiang et al., 2019), thereby affecting various tumor biological processes such as proliferation, drug resistance, and tumor metabolism (Dang et al., 2014; Liu et al., 2019). For example, circCCDC66 in colorectal cancer can sponge miR-33b/miR-93 and prevent c-Myc mRNA degradation, thereby promoting cancer cell proliferation and tumor growth (Zeng et al., 2016). In triple-negative breast cancer, circANKS1B massively absorbs miR-148a-3p/miR-152-3p to increase USF1 expression and then drives the epithelial-mesenchymal transition (EMT) process (Guo et al., 2020). circMine (http://hpcc.siat.ac.cn/circmine/home) database analysis revealed increased expression of hsa_circ_0006877 (labeled as circLDLR, derived from the low-density lipoprotein receptor provided by HGNC gene) expression increased in GC. Significantly, it has been recognized that circLDLR mediates disease progression in various cancers such as colorectal cancer, thyroid cancer, and polyovarian syndrome (DeBerardinis, 2008; Ocampo et al., 2016; Han et al., 2020).

miRNAs can not only form a complex biological process regulation network with ncRNAs but also directly bind to target mRNAs to negatively regulate gene expression at the transcriptional level (Sexton et al., 2020). Based on this, the downstream miRNA of circLDLR and target genes of miRNA, specifically miR-449b-5p and CHD1, were screened. miR-449b-5p is recognized as a tumor suppressor that regulates tumor proliferation, metastasis, and invasion (Garber, 2004; Chen et al., 2020; Huang et al., 2020). CHD1, a regulator in maintaining genome stability, promotes DNA transcription by keeping DNA in an open and transcriptionally active state through ATP-dependent assembly, transfer, and removal of nucleosomes from DNA (Li et al., 2020; Qiu et al., 2022). It has been emphasized that CHD1 degradation suppresses glioma cell proliferation and glycolysis (Jeck et al., 2013).

The current study explored and analyzed the biological function of circLDLR in GC at the molecular level and defined the interlink between circLDLR, miR-449b-5p, and CHD1 to develop a theoretical reference to control GC progression.

## 2. Materials and methods

### 2.1. Clinical samples

Forty-three pairs of tumor specimens and adjacent normal tissue samples were collected from GC patients, and all tissues were rapidly cryopreserved in liquid nitrogen. Written informed consent was obtained from each patient. This study was approved by the Institutional Review Board of Affiliated Hospital of Traditional Chinese Medicine of Xinjiang Medical University.

### 2.2. Cell culture

The GC cell lines (MKN-45 and AGS) were purchased from Procell (Wuhan, China), and the human gastric mucosa cell line GES-1 was from COBIOER Bioscience (Nanjing, China). Under standard conditions (37 °C + 5% CO_2_) MKN-45 and GES-1 cells were cultured in RPMI-1640 medium, while AGS cells were in Ham’s F-12 medium. Each medium was supplemented with 10% fetal bovine serum and 1% penicillin-streptomycin (Procell).

### 2.3. Cell transfection

The circLDLR siRNA (si-circLDLR), CHD1 siRNA (si-CHD1), miR-449b-5p mimic, and GV485-constructed circLDLR overexpression plasmid (CirclDLR-OE), along with their corresponding negative controls, were synthesized by GenePharma (Shanghai, China). In MKN-45 cells, transfection with the above oligonucleotide and vector was constructed using Lipofectamine 2000 (Invitrogen) and lasted for 48 h until checking for the transfection efficiency by quantitative PCR and immunoblot analysis. The experiments were performed in 3 biological repetitions.

### 2.4. Quantitative PCR

To generate total RNA from tissues and cells, TRIzol reagent (Invitrogen) was applied. Subsequently, RNA samples were checked by Nanodrop 2000 (Thermo Fisher Scientific) to determine the purity and concentration. Following this, 1.5 μg of mRNA and circRNA were reverse transcribed into cDNA using SuperScriptTM III Reverse Transcriptase (Invitrogen) and subjected to PCR detection using SYBR Green master mix (CloudSeq). miRNA amplification was carried out using TaqMan MicroRNA Reverse Transcription Kit (Applied Biosystems) and TaqMan Universal Master Mix II (Applied Biosystems), followed by PCR detection in the Applied Biosystems AB7500 Real-Time PCR system. To calculate gene expression which is normalized to U6 (for miR-449b-5p) or GAPDH (for circLDLR and CHD1), the 2^−^^ΔΔ^^CT^ method was employed. Three biological replicates were performed for each experiment. The primers are shown in Table 1.

### 2.5. RNAse R treatment

After the total RNA (10 μg) of MKN-45 cells was co-incubated with RNAse R (3 U/g, Epicentre Technologies, USA) at 37°C for 30 min, the stability of circLDLR and LDLR mRNA was detected by quantitative PCR.

### 2.6. Actinomycin D assay

MKN-45 cells on the 6-well plates (5 × 10^5^ cells/well) were treated with 2 μg/mL of actinomycin D (MCE, HY-17559), and detected at 0, 4, 8, 12, and 24 h, respectively. Linear and circular LDLR mRNA content was analyzed by quantitative PCR and normalized to the measured value at 0 h.

### 2.7. Subcellular isolation assay

A nucleoplasmic fractionation assay was performed using PARIS Kit (Invitrogen). MKN-45 cells (1 × 10^6^) were collected in a cell separation buffer and centrifuged to separate the nuclei and cytoplasm. Gene expression was then measured using quantitative PCR, with 18S serving as the cytoplasmic reference and U6 as the nuclear reference.

### 2.8. Immunoblot analysis

Cells were lysed by adding lysis buffer (Beyotime, China) for 20 min on ice, and the protein concentration was measured using the Bradford assay (Bio-Rad, USA). Next, proteins subjected to 15% SDS-PAGE were loaded onto PVDF membranes, which were then blocked with 5% nonfat milk powder for 1 h and reacted with primary antibodies GAPDH (2118, CST, USA) and CHD1 (4351, CST, USA) overnight at 4 °C. After TBST washing, the cells were incubated with the corresponding horseradish peroxidase-conjugated secondary antibody (CST, USA) for 1 h at 37 °C and visualized using an enhanced ECL chemiluminescence kit (ultrassignal, China).

### 2.9. CCK-8

MKN-45 cell suspension (100 μL) was placed in 96-well plate at 3 × 10^3^ cells/well to calculate cell proliferation at 0, 24, 48, and 72 h. At each time point, 10 μL of CCK-8 solution (Dojindo, Japan) was added and incubated at 37 °C for 2 h. Absorbance at 450 nm was then measured on a microplate reader (Bio-Rad).

### 2.10. EdU experiment

The EdU assay was performed using BeyoClick EdU Cell Proliferation Kit and Alexa Fluor 555 (Beyotime). MKN-45 cells (1 × 10^5^) were covered over 15 mm dishes overnight, treated with a 10-μM EdU solution for 2 h, fixed with 4% paraformaldehyde, permeated with 0.3% Triton X-100, and washed with 3% BSA. Finally, the cells were treated with Click Additive Solution and Hoechst 33342 and viewed under a confocal laser scanning microscope (Olympus FLUOVIEW FV1000).

### 2.11. Glucose uptake and lactate production

Glucose uptake analysis was performed using a glucose detection kit (BioVision, Milpitas, CA, USA). MKN-45 cells were incubated in 6-well plates at a density of 1 × 10^6^/well for 48 h. Glucose uptake = (0-h concentration − 48-h concentration)/protein concentration.

Lactate content in the culture medium was measured using a lactate detection kit (BioVision) and normalized to protein concentrations.

### 2.12. ATP/ADP ratio

ATP/ADP was measured using ApoSENSOR ADP/ATP ratio assay kit (BioVision), and luminescence was measured at SpectraMax (Molecular Devices, USA). MKN-45 cells (1 × 10^4^) were mixed with the nucleotide release buffer, followed by 1 μL of ATP monitoring enzyme. Luminescence readings at 1 min (Data A) and 10 min (Data B) were recorded. Data C was determined after adding ADP converting enzyme. The ATP/ADP = Data A/(Data C − Data B).

### 2.13 NAD^+^/NADH ratio

NAD^+^/NADH was determined using an EnzyLight NAD^+^/NADH ratio assay kit (Bioassay Systems, USA). MKN-45 cells were resuspended with 100 μL of NAD^+^ extraction buffer to determine NAD^+^. For NADH determination, 100 μL of NADH extraction buffer was added. After heating at 60°C for 5 min, assay buffer (20 μL) was added to the extract, followed by centrifugation and addition of working reagent to the resulting supernatant. Absorbance at 565 nm was read at 0 and 15 min.

### 2.14. Apoptosis assay

The apoptosis rate was detected using the Annexin V-FITC/PI Apoptosis Detection Kit (Solarbio, CA1020). MKN-45 cells were washed with precooled PBS and resuspended in 1 mL of 1X binding buffer. The cell suspension (100 μL, 1 × 10^6^ cells/mL) was mixed with 5 μL of Annexin V-FITC for 10 min, followed by 5 μL of propidium iodide for 5 min. Data were acquired on a FACScan flow cytometer (BD Biosciences, USA).

### 2.15. Luciferase reporter assay

To predict binding sites between miR-449b-5p and circLDLR or CHD1, starBase 3.0 (http://starbase.sysu.edu.cn/) was utilized. The binding region was mutated and cloned into the pmirGLO luciferase reporter vector (Promega) to generate WT-circLDLR, MUT-circLDLR, WT-CHD1, and MUT-CHD1, respectively. All plasmids were synthesized by Genepharma (Shanghai, China). Luciferase reporters were cotransfected into MKN-45 cells with either miR-449b-5p mimic or mimic NC using Lipofectamine 2000 (Invitrogen). After 48 h of incubation, luciferase activity was measured using a dual luciferase reporter assay kit (Promega) on a Synergy 2 Multidetector Microplate Reader (BioTek. USA).

### 2.16. RNA binding protein immunoprecipitation (RIP) experiment

Cell lysates were prepared using RNA immunoprecipitation lysis buffer supplemented with protease inhibitors and RNAse inhibitors, and then mixed with magnetic bead-conjugated Ago2 or anti-IgG for 6 h at 4 °C. After elution, the immunoprecipitates were purified and analyzed by quantitative PCR. The necessary materials for this procedure were obtained from the EZ-Magna RIP kit (Millipore).

### 2.17. Xenotransplantation and immunohistochemistry

The animal experiment was approved by the Animal Ethics Committee of Affiliated Hospital of Traditional Chinese Medicine of Xinjiang Medical University. BALB/c female nude mice (6 weeks old; obtained from Shanghai Experimental Animal Research Center) were randomly divided into two groups, with 4 mice in each group. MKN-45 cells (5 × 10^6^ cells) transfected with si-circLDLR were injected subcutaneously into the right abdominal muscle layer of the mice. Tumor diameter was measured every 7 days to calculate tumor volume (1/2 × tumor length × tumor width^2^). At the end of week 4, euthanasia was performed to harvest the tumors. The tumors were fixed with 10% formalin and prepared into paraffin sections (5 μm) for immunohistochemical analysis of Ki-67 (ab15580, Abcam).

### 2.18. Data analysis

The data are presented as mean ± standard deviation (SD). Student’s t-test was used for comparisons between two groups, while one-way analysis of variance (ANOVA) was employed for comparisons among multiple groups. Each experiment was conducted with at least three biological replicates. A p-value <0.05 was considered statistically significant.

## 3. Results

### 3.1. CircLDLR expression signature in GC

**Figure 1:**
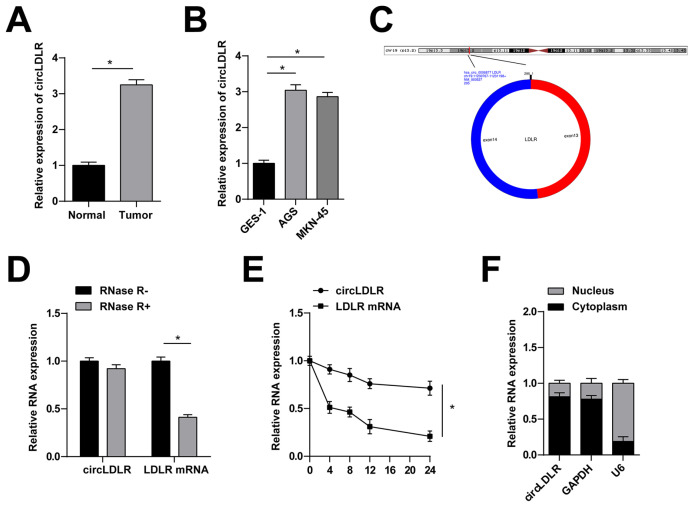
CircLDLR expression signature in GC. A: quantitative PCR to detect circLDLR in GC tissues and adjacent normal tissues. B: quantitative PCR to detect circLDLR in human normal gastric mucosa cells GES-1 and human GC cells AGS and MKN-45. C: circLDLR gene information. D: RNAse R experiment to verify the ring structure of circLDLR. E: actinomycin D experiment to verify the stability of circLDLR. F: Subcellular isolation experiment to detect circLDLR localization in MKN-45 cells. Data were expressed as mean ± SD (repetitions = 3)

Quantitative PCR analysis demonstrated higher levels of circLDLR in GC tissues compared to normal tissues (Figure 1A), as well as in AGS and MKN-45 cells compared to GES-1 cells (Figure 1B). The correlation between circLDLR and clinicopathological features of GC patients was clarified. The results revealed that high expression of circLDLR was correlated with advanced tumor stage and poor differentiation of TNM (Table 2).

Using the bioinformatics website circBank (http://www.circbank.cn/), it was determined that circLDLR has a predicted length of 295nt and originates from exons 13 and 14 of the LDLR gene (Figure 1C). Testing the stability of circLDLR using RNAse R and actinomycin D assays revealed that circLDLR was resistant to RNAse R digestion and exhibited a longer half-life compared to linear RNA (Figures 1D and 1E). In addition, subcellular isolation experiments confirmed the main localization of circLDLR in the cytoplasm of MKN-45 cells (Figure 1F).

### 3.2. circLDLR induces proliferation and aerobic glycolysis of GC cells

**Figure 2:**
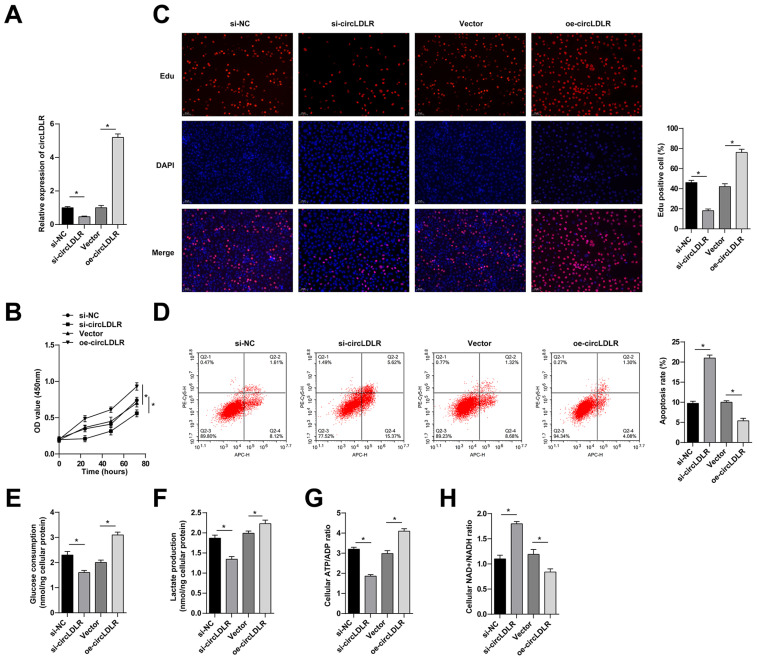
circLDLR induces proliferation and aerobic glycolysis of GC cells. MKN-45 cells were transfected with si-circLDLR or circlDLR-OE. A: quantitative PCR to detect circLDLR and LDLR. B: CCK-8 assay to detect the proliferation of MKN-45 cells. C: EdU assay to detect the proliferation ability of MKN-45 cells. D: Flow cytometry to detect apoptosis of MKN-45 cells. E: glucose uptake. F: lactic acid production. G: ATP/ADP ratio. H: NAD^+^/NADH ratio. Data were expressed as mean ± SD (repetitions = 3).

circLDLR gain- and loss-of-function assays were conducted by transfecting circLDLR-oe and si-circLDLR into MKN-45 cells, respectively, and quantitative PCR validated that circLDLR in the cells was stably upregulated or downregulated (Figure 2A). CCK-8 assay and EdU assay manifested that silencing circLDLR was suppressive for the proliferative activity of MKN-45 cells (Figures 2B and 2C). Moreover, flow cytometry revealed that depletion of circLDLR promoted apoptosis in MKN-45 cells (Figure 2D). In addition, circLDLR deficiency in MKN-45 cells lowered glucose uptake, lactate production, ATP/ADP ratio, and increased NAD^+^/NADH ratio (Figures 2E–2H). Consistent with the above results, when circLDLR was overexpressed, cell proliferation and aerobic glycolysis were enhanced, while apoptosis was disrupted.

### 3.3. circLDLR sponges miR-449b-5p in GC cells

**Figure 3:**
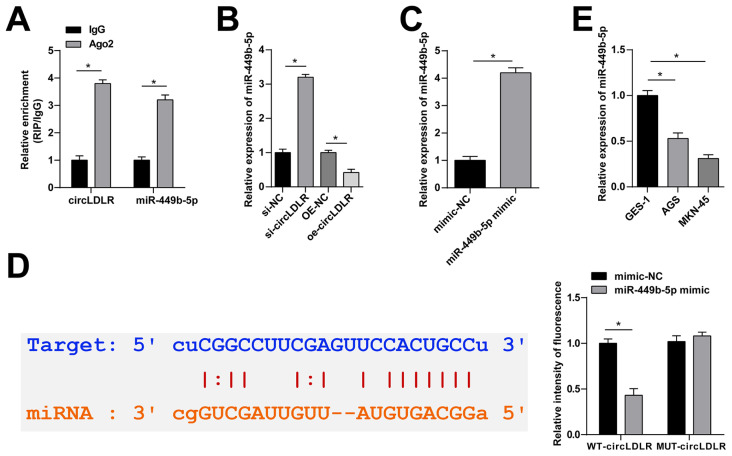
circLDLR sponges miR-449b-5p in GC cells. A: RIP assay to detect the enrichment of circLDLR and miR-449b-5p in MKN-45 cells. B: quantitative PCR to detect miR-449b-5p expression. C: quantitative PCR to detect miR-449b-5p in MKN-45 cells after transfection with miR-449b-5p mimic. D: luciferase reporter assay to analyze the targeting relationship between circLDLR and miR-449b-5p. E: quantitative PCR to detect miR-449b-5p in GC cells. Data were expressed as mean ± SD (repetitions = 3).

Potential miRNAs of circLDLR were predicted in the starBase3.0 database, and 5 tumor-related miRNAs were screened out. RIP experimental results emphasized that miR-449b-5p and circLDLR were enriched in Ago2 (Figure 3A). In MKN-45 cells altering circLDLR, quantitative PCR noted that si-circLDLR and circLDLR-oe increased and suppressed miR-449b-5p, respectively (Figure 3B). In addition, quantitative PCR detected miR-449b-5p in MKN-45 cells after transfection with miR-449b-5p mimic, and confirmed the upregulation efficacy on miR-449b-5p expression (Figure 3C). As determined by luciferase reporter assay, miR-449b-5p upregulation impaired the luciferase activity of WT-circLDLR reporter but not MUT-circLDLR (Figure 3D). Notably, miR-449b-5p was expressed highly in AGS and MKN-45 cells (Figure 3E).

### 3.4. circLDLR/miR-449b-5p interlink affects the proliferation and aerobic glycolysis of GC cells

**Figure 4:**
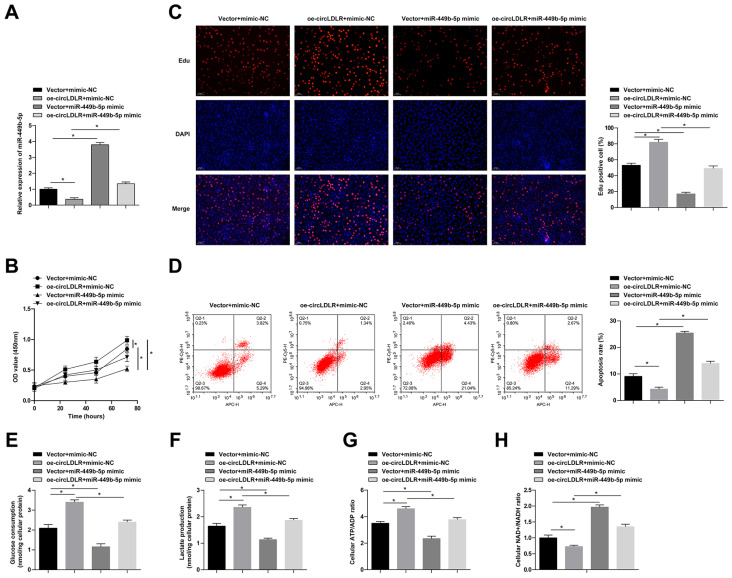
circLDLR/miR-449b-5p interlink affects the proliferation and aerobic glycolysis of GC cells. circLDLR-oe and miR-449b-5p mimic were cotransfected into MKN-45 cells. A: quantitative PCR to detect miR-449b-5p. B: CCK-8 assay to detect the proliferation of MKN-45 cells. C: EdU assay to detect the proliferation ability of MKN-45 cells. D: Flow cytometry to detect apoptosis of MKN-45 cells. E: glucose uptake. F: lactic acid production. G: ATP/ADP ratio. H: NAD^+^/NADH ratio. Data were expressed as mean ± SD (repetitions = 3).

A rescue experiment was designed by cotransfecting circlDLR-OE and miR-449b-5p mimic into MKN-45 cells. Quantitative PCR analysis revealed that the miR-449b-5p mimic reactivated miR-449b-5p expression that had been suppressed by circLDLR-oe (Figure 4A). Subsequently, the rescue effects of miR-449b-5p reactivation on proliferation, apoptosis, and aerobic glycolysis were observed in MKN-45 cells overexpressing circLDLR (Figures 4B–4H).

### 3.5. miR-449b-5p degrades CHD1

**Figure 5:**
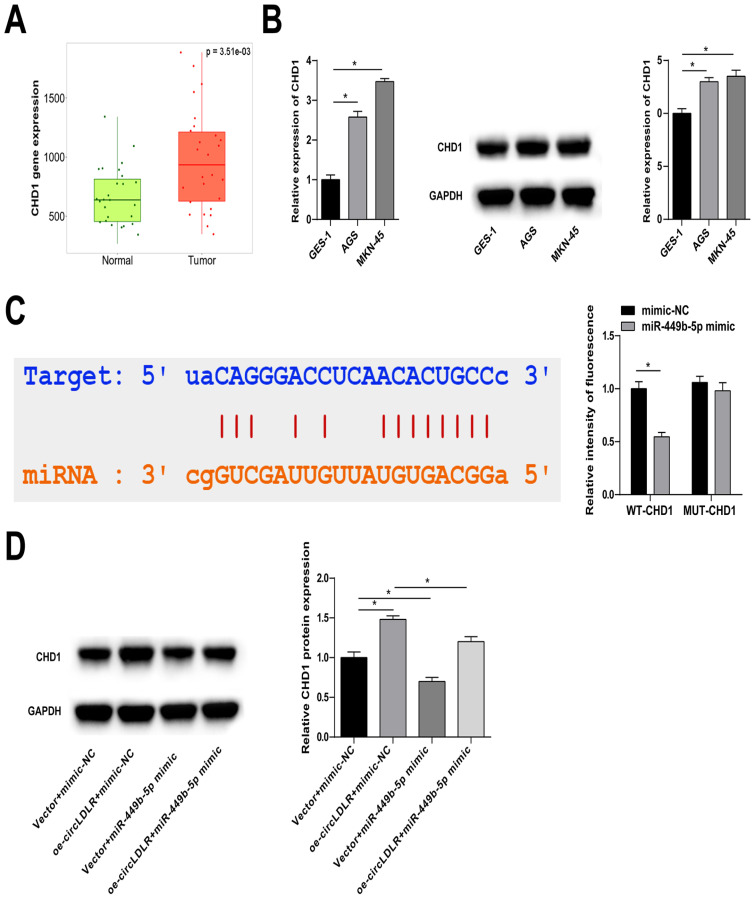
miR-449b-5p degrades CHD1. A: TNMplot.com database to analyze the expression patterns of target genes in GC and normal tissues. B: quantitative PCR and immunoblot analysis to detect CHD1 in GC cells. C: luciferase reporter assay to detect the interaction between CHD1 and miR-449b-5p. D: immunoblot analysis to detect CHD1 in MKN-45 cells. Data were expressed as mean ± SD (repetitions = 3).

Potential target proteins of miR-449b-5p were initially predicted using the starBase 3.0 database, and their gene expression patterns were analyzed at TNMplot.com (https://tnmplot.com/analysis/). Subsequently, it was observed that CHD1 expression was abnormally increased in GC (Figure 5A). Quantitative PCR and immunoblot analysis confirmed the upregulation of CHD1 in AGS and MKN-45 cells (Figure 5B). Furthermore, the targeting relationship between CHD1 and miR-449b-5p in MKN-45 cells was confirmed by a dual luciferase reporter assay (Figure 5C). Moreover, in MKN-45 cells, it was observed that circLDLR-oe and miR-449b-5p mimic augmented and limited CHD1 expression, respectively. However, miR-449b-5p mimic cotransfection weakened circLDLR-oe-induced CHD1 upregulation (Figure 5D).

### 3.6. circLDLR/miR-449b-5p/CHD1 feedback in the malignant progression of GC cells

**Figure 6:**
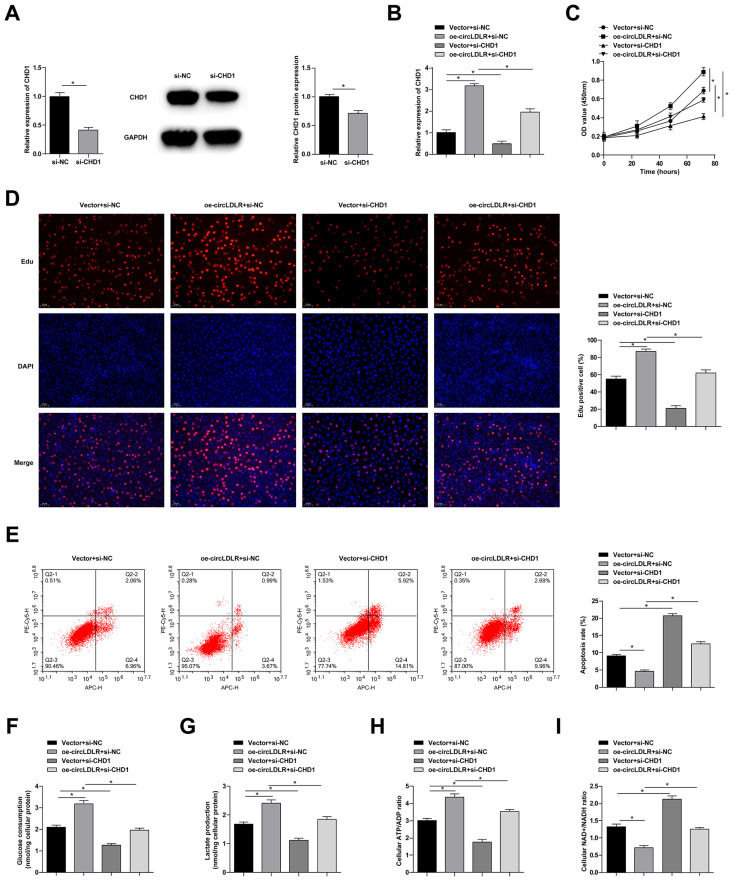
circLDLR/miR-449b-5p/CHD1 feedback in the malignant progression of GC cells. A: si-CHD1 was transfected into MKN-45 cells, quantitative PCR and immunoblot analysis to detect CHD1. B: circLDLR-oe and si-CHD1 were cotransfected into MKN-45 cells, quantitative PCR to detect CHD1. C: CCK-8 assay to detect the proliferation of MKN-45 cells. D: EdU assay to detect the proliferation ability of MKN-45 cells. E: Flow cytometry to detect apoptosis of MKN-45 cells. F: glucose uptake. G: lactic acid production. H: ATP/ADP ratio. I: NAD^+^/NADH ratio. Data were expressed as mean ± SD (repetitions = 3).

si-CHD1 was transfected into MKN-45 cells, and quantitative PCR and immunoblot analysis validated the knockdown efficiency of CHD1 (Figure 6A). In the meantime, circLDLR-OE and si-CHD1 were cotransfected into MKN-45 cells, and quantitative PCR assay confirmed that si-CHD1 lowered CHD1 levels based on circLDLR-oe (Figure 6B). Subsequent cell assays revealed that silencing CHD1 mitigated circLDLR-oe-resulted trends of proliferation, apoptosis, and aerobic glycolysis in GC cells (Figure 6C–6I).

### 3.7. Tumor growth mediated by circLDLR in vivo

**Figure 7:**
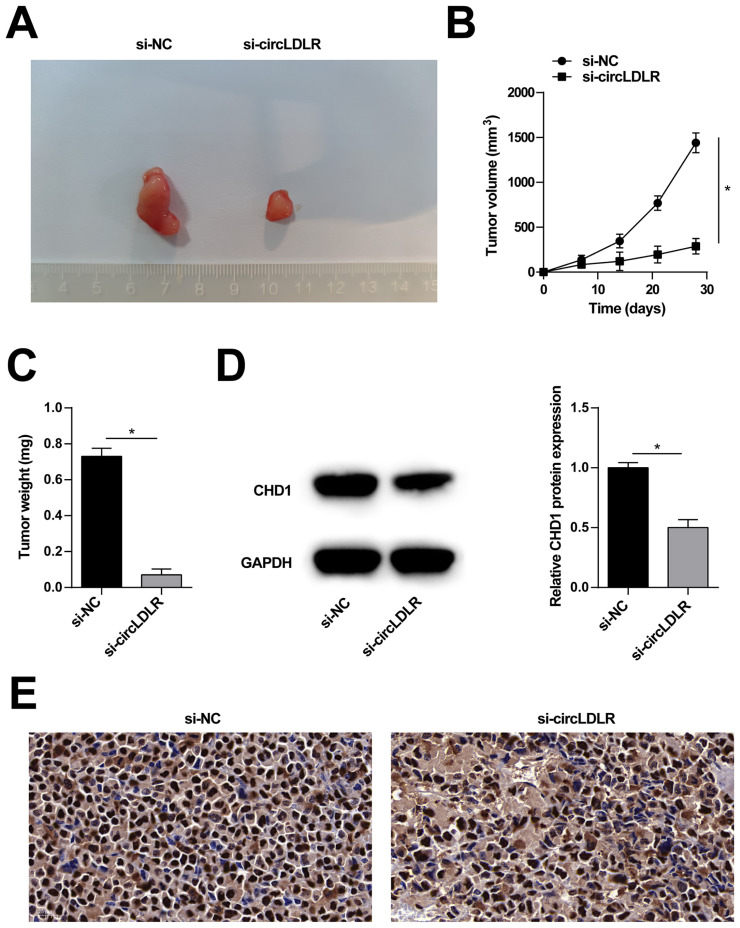
Tumor growth mediated by circLDLR in vivo. A: Representative image of the tumor. B: Growth curve of tumors. C: Tumor weight. D: immunoblot analysis to detect CHD1 in xenograft tumors. E: Immunohistochemical analysis to detect Ki-67 in xenograft tumors. Data were expressed as mean ± SD (n = 4).

After xenotransplantation with MKN-45 cells, tumors developed in nude mice. Interestingly, circLDLR knockdown impaired the tumorigenic ability of MKN-45 cells, as evidenced by reductions in tumor volume and weight (Figures 7A–7C). Additionally, circLDLR knockdown led to decreased expression of CHD1 protein (Figure 7D) and reduced levels of Ki-67 in the tumors (Figure 7E).

## 4. Discussion

Due to the complex and heterogeneous nature of GC, progress has been limited to control tumor development. CircRNAs are widely and abnormally expressed in tumors, including GC, and indicate an association with tumor progression (Memczak et al., 2013; Zheng et al., 2016; Wang et al., 2019). Particularly, the injection of synthetic circRNA into patients may be an effective approach for future cancer treatment (Wang et al., 2022). In pursuit of potential regulators of GC, the current study evaluated circLDLR on proliferation, apoptosis, and aerobic glycolysis of GC, and eventually confirmed that circLDLR/miR-449b-5p/CHD1 feedback aggravates GC malignancy.

CircLDLR is transcriptively processed from its parent LDLR and is involved in ovarian steroidogenesis to mediate follicular dysplasia in polycystic ovary syndrome. Furthermore, a recent report has mentioned that abundant circLDLR is closely related to the malignant progression of colorectal cancer cells. Based on this, the current study focused on circLDLR-related mechanisms in GC. It was found that circLDLR was stably and highly expressed in GC, mainly in the cytoplasm of GC cells. Furthermore, silencing circLDLR inhibited GC tumor growth; overexpressing circLDLR stimulated the proliferative and antiapoptotic abilities of MKN-45 cells while silencing circLDLR had the opposite effect. Considering that aerobic glycolysis is a hallmark in the development of GC (Luo et al., 2020; Pu et al., 2020), aerobic glycolysis-related indicators were tested, finally confirming the strengthening effect of circLDLR overexpression on aerobic glycolysis and the inhibitory effect of circLDLR knockdown.

CircRNAs can participate in biological processes through various mechanisms (Holdt et al., 2016; Huang et al., 2019). Among them, the main biological function of circRNA in tumors is to promote miRNA degradation by binding with miRNA (Meng et al., 2022). At present, studies on circLDLR have confirmed that it affects papillary thyroid cancer and colorectal cancer as ceRNA sponge miRNA. Similarly, the current study confirmed the targeting relationship between circLDLR and miR-449b-5. It has been discussed that miR-449b-5p blocks the malignant behaviors of cancer cells, thereby regulating tumor progression in various cancers including glioma, colorectal cancer, and lung adenocarcinoma (Hall and Georgel, 2007; Marfella et al., 2007; Jiang et al., 2021). In the setting of GC, the current study measured miR-449b-5p downregulation in tumor cells and identified that miR-449b-5p upregulation counteracted proliferation, apoptosis, and aerobic glycolysis mediated by circLDLR overexpression.

Subsequently, CHD1, an upregulated protein in GC, was screened out as a target of miR-449b-5p. Classified as a member of the CHD family, CHD1 is involved in transcription, replication, recombination, and DNA repair processes (Salzman et al., 2013; Kristensen et al., 2019). In tumor progression, CHD1 has been considered to accelerate glioma growth in the network of circRNA and miRNA. The current study elaborated that CHD1 was coregulated by circLDLR and miR-449b-5p, and furthermore, CHD1 deficiency attenuated the tumor-promoting effects induced by circLDLR in GC.

Despite these insights, there are limitations to be addressed in subsequent experiments. Future studies will delve into the molecular mechanisms by which circLDLR mediates the miR-449b-5p/CHD1 axis to affect the proliferation and aerobic glycolysis of GC cells, including its interactions with glycolytic genes and signaling pathways. Moreover, additional clinical samples should be collected for verification and experimental purposes.

## 5. Conclusion

CircLDLR in GC can target CHD1 through miR-449b-5p, and then participates in the proliferation and aerobic glycolysis of GC cells, which provides a new target and reference for the clinical treatment of GC patients.

## Figures and Tables

**Table 1:** PCR primers.

Genes	PCR primer sequences (5′– 3′)
circLDLR	Forward: AGCTGCCTCACAGGACAAAG
	Reverse: GGTCCTCTCACACCAGTTCAC
miR-449b-5p	Forward: GCGCGGCAGTGTATTGTTA
	Reverse: GCAGGGTCCGAGGTATTC
CHD1	Forward: TGTAGCCCTGAAGAAGCAGC
	Reverse: AAGGCCCATTTCATCAGCGA
U6	Forward: CTCGCTTCGGCAGCACA
	Reverse: AACGCTTCACGAATTTGCGT
GAPDH	Forward: CACCCACTCCTCCACCTTTG
	Reverse: CCACCACCCTGTTGCTGTAG

Note: circLDLR, circular RNA LDLR; miR-449b-5p, microRNA-449b-5p; CHD1, Chromohelicase/ATPase DNA-binding protein 1; GAPDH, glyceraldehyde-3phosphate dehydrogenase.

**Table 2:** Association between circLDLR expression and clinicopathological characteristics of gastric cancer.

Characteristics	circLDLR expression			p
	Low (n = 21)	High (n = 22)		
Age (years)				0.9065
<60	13	14	27	
≥60	8	8	16	
Sex				0.4383
Male	14	17	31	
Female	7	5	12	
T classification				0.8992
T1	5	4	9	
T2	6	7	13	
T3	10	11	21	
N classification				0.8661
N0	9	11	20	
N1	7	6	13	
N2	4	3	7	
N3	1	2	3	
TNM stage				0.0096
I+II	14	6	20	
III+IV	7	16	23	
Differentiation grade				0.0082
G1+G2	12	4	16	
G3	9	18	27	

Chi-squared test. p < 0.05 was considered statistically significant.

## Data Availability

The datasets used and/or analyzed during the present study are available from the corresponding author upon reasonable request.

## References

[b1-tjb-48-01-046] Chen LL 2020 The expanding regulatory mechanisms and cellular functions of circular RNAs Nature reviews Molecular Cell Biology 21 8 475 490 10.1038/s41580-020-0243-y 32366901

[b2-tjb-48-01-046] Cheng L Shi X Huo D Zhao Y Zhang H 2019 MiR-449b-5p regulates cell proliferation, migration and radioresistance in cervical cancer by interacting with the transcription suppressor FOXP1 European Journal of Pharmacology 856 172399 10.1016/j.ejphar.2019.05.028 31103631

[b3-tjb-48-01-046] Dang Y Wang YC Huang QJ 2014 Microarray and next-generation sequencing to analyse gastric cancer Asian Pacific Journal of Cancer Prevention: APJCP 15 19 8033 9 25338980

[b4-tjb-48-01-046] DeBerardinis RJ 2008 Is cancer a disease of abnormal cellular metabolism? New angles on an old idea Genetics in Medicine: Official Journal of the American College of Medical Genetics 10 11 767 77 10.1097/GIM.0b013e31818b0d9b 18941420 PMC2782690

[b5-tjb-48-01-046] Garber K 2004 Energy boost: the Warburg effect returns in a new theory of cancer Journal of the National Cancer Institute 96 24 1805 6 10.1093/jnci/96.24.1805 15601632

[b6-tjb-48-01-046] Guo K Gong W Wang Q Gu G Zheng T 2020 LINC01106 drives colorectal cancer growth and stemness through a positive feedback loop to regulate the Gli family factors Cell Death & Disease 11 10 869 10.1038/s41419-020-03026-3 33067422 PMC7567881

[b7-tjb-48-01-046] Hall JA Georgel PT 2007 CHD proteins: a diverse family with strong ties Biochemistry and cell biology = Biochimie et Biologie Cellulaire 85 4 463 76 10.1139/o07-063 17713581

[b8-tjb-48-01-046] Han C Wang S Wang H Zhang J 2020 Knockdown of circ-TTBK2 Inhibits Glioma Progression by Regulating miR-1283 and CHD1 Cancer Management and Research 12 10055 10065 10.2147/cmar.s252916 33116862 PMC7568596

[b9-tjb-48-01-046] Hansen TB Jensen TI Clausen BH Bramsen JB Finsen B 2013 Natural RNA circles function as efficient microRNA sponges Nature 495 7441 384 8 10.1038/nature11993 23446346

[b10-tjb-48-01-046] Holdt LM Stahringer A Sass K Pichler G Kulak NA 2016 Circular non-coding RNA ANRIL modulates ribosomal RNA maturation and atherosclerosis in humans Nature Communications 7 12429 10.1038/ncomms12429 PMC4992165 27539542

[b11-tjb-48-01-046] Hsiao KY Lin YC Gupta SK Chang N Yen L 2017 Noncoding Effects of Circular RNA CCDC66 Promote Colon Cancer Growth and Metastasis Cancer Research 77 9 2339 2350 10.1158/0008-5472.can-16-1883 28249903 PMC5910173

[b12-tjb-48-01-046] Huang X Li Z Zhang Q Wang W Li B 2019 Circular RNA AKT3 upregulates PIK3R1 to enhance cisplatin resistance in gastric cancer via miR-198 suppression Molecular Cancer 18 1 71 10.1186/s12943-019-0969-3 30927924 PMC6441201

[b13-tjb-48-01-046] Huang X Wu B Chen M Hong L Kong P 2020 Depletion of exosomal circLDLR in follicle fluid derepresses miR-1294 function and inhibits estradiol production via CYP19A1 in polycystic ovary syndrome Aging 12 15 15414 15435 10.18632/aging.103602 32651991 PMC7467373

[b14-tjb-48-01-046] Jeck WR Sorrentino JA Wang K Slevin MK Burd CE 2013 Circular RNAs are abundant, conserved, and associated with ALU repeats RNA (New York, NY) 19 2 141 57 10.1261/rna.035667.112 PMC3543092 23249747

[b15-tjb-48-01-046] Jiang J Yang X He X Ma W Wang J 2019 MicroRNA-449b-5p suppresses the growth and invasion of breast cancer cells via inhibiting CREPT-mediated Wnt/β-catenin signaling Chemico-Biological Interactions 302 74 82 10.1016/j.cbi.2019.02.004 30738779

[b16-tjb-48-01-046] Jiang YM Liu W Jiang L Chang H 2021 CircLDLR Promotes Papillary Thyroid Carcinoma Tumorigenicity by Regulating miR-637/LMO4 Axis Disease Markers 2021 3977189 10.1155/2021/3977189 PMC8677406 34925640

[b17-tjb-48-01-046] Kristensen LS Andersen MS Stagsted LVW Ebbesen KK Hansen TB 2019 The biogenesis, biology and characterization of circular RNAs Nature Reviews Genetics 20 11 675 691 10.1038/s41576-019-0158-7 31395983

[b18-tjb-48-01-046] Li J Sun D Pu W Wang J Peng Y 2020 Circular RNAs in Cancer: Biogenesis, Function, and Clinical Significance Trends in Cancer 6 4 319 336 10.1016/j.trecan.2020.01.012 32209446

[b19-tjb-48-01-046] Liu X Abraham JM Cheng Y Wang Z Wang Z 2018 Synthetic Circular RNA Functions as a miR-21 Sponge to Suppress Gastric Carcinoma Cell Proliferation Molecular Therapy Nucleic Acids 13 312 321 10.1016/j.omtn.2018.09.010 30326427 PMC6197335

[b20-tjb-48-01-046] Liu X Wang X Zhang J Lam EK Shin VY 2010 Warburg effect revisited: an epigenetic link between glycolysis and gastric carcinogenesis Oncogene 29 3 442 50 10.1038/onc.2009.332 19881551

[b21-tjb-48-01-046] Liu Y Zhang Z Wang J Chen C Tang X 2019 Metabolic reprogramming results in abnormal glycolysis in gastric cancer: a review OncoTargets and Therapy 12 1195 1204 10.2147/ott.s189687 30863087 PMC6389007

[b22-tjb-48-01-046] Luo Z Rong Z Zhang J Zhu Z Yu Z 2020 Circular RNA circCCDC9 acts as a miR-6792-3p sponge to suppress the progression of gastric cancer through regulating CAV1 expression Molecular Cancer 19 1 86 10.1186/s12943-020-01203-8 32386516 PMC7210689

[b23-tjb-48-01-046] Marfella CG Imbalzano AN 2007 The Chd family of chromatin remodelers Mutation Research 618 1–2 30 40 10.1016/j.mrfmmm.2006.07.012 17350655 PMC1899158

[b24-tjb-48-01-046] Memczak S Jens M Elefsinioti A Torti F Krueger J 2013 Circular RNAs are a large class of animal RNAs with regulatory potency Nature 495 7441 333 8 10.1038/nature11928 23446348

[b25-tjb-48-01-046] Meng L Zhang Y Wu P Li D Lu Y 2022 CircSTX6 promotes pancreatic ductal adenocarcinoma progression by sponging miR-449b-5p and interacting with CUL2 Molecular Cancer 21 1 121 10.1186/s12943-022-01599-5 35650603 PMC9158112

[b26-tjb-48-01-046] Ocampo J Chereji RV Eriksson PR Clark DJ 2016 The ISW1 and CHD1 ATP-dependent chromatin remodelers compete to set nucleosome spacing in vivo Nucleic Acids Research 44 10 4625 35 10.1093/nar/gkw068 26861626 PMC4889916

[b27-tjb-48-01-046] Pu Z Xu M Yuan X Xie H Zhao J 2020 Circular RNA circCUL3 Accelerates the Warburg Effect Progression of Gastric Cancer through Regulating the STAT3/HK2 Axis Molecular Therapy Nucleic Acids 22 310 318 10.1016/j.omtn.2020.08.023 33230436 PMC7527579

[b28-tjb-48-01-046] Qiu Y Chen Y Agbede O Eshaghi E Peng C 2022 Circular RNAs in Epithelial Ovarian Cancer: From Biomarkers to Therapeutic Targets Cancers 14 22 10.3390/cancers14225711 PMC9688053 36428803

[b29-tjb-48-01-046] Salzman J Chen RE Olsen MN Wang PL Brown PO 2013 Cell-type specific features of circular RNA expression PLoS Genetics 9 9 e1003777 10.1371/journal.pgen.1003777 24039610 PMC3764148

[b30-tjb-48-01-046] Sexton RE Al Hallak MN Diab M Azmi AS 2020 Gastric cancer: a comprehensive review of current and future treatment strategies Cancer Metastasis Reviews 39 4 1179 1203 10.1007/s10555-020-09925-3 32894370 PMC7680370

[b31-tjb-48-01-046] Skene PJ Hernandez AE Groudine M Henikoff S 2014 The nucleosomal barrier to promoter escape by RNA polymerase II is overcome by the chromatin Chd1 eLife 3 e02042 10.7554/eLife.02042 24737864 PMC3983905

[b32-tjb-48-01-046] Vander Heiden MG Cantley LC Thompson CB 2009 Understanding the Warburg effect: the metabolic requirements of cell proliferation Science (New York, NY) 324 5930 1029 33 10.1126/science.1160809 PMC2849637 19460998

[b33-tjb-48-01-046] Vasudevan S Tong Y Steitz JA 2007 Switching from repression to activation: microRNAs can up-regulate translation Science (New York, NY) 318 5858 1931 4 10.1126/science.1149460 18048652

[b34-tjb-48-01-046] Wang R Wang J Chen Y Chen Y Xi Q 2022 Circular RNA circLDLR facilitates cancer progression by altering the miR-30a-3p/SOAT1 axis in colorectal cancer Cell Death Discovery 8 1 314 10.1038/s41420-022-01110-5 35821230 PMC9276972

[b35-tjb-48-01-046] Wang S Zhang Y Cai Q Ma M Jin LY 2019 Circular RNA FOXP1 promotes tumor progression and Warburg effect in gallbladder cancer by regulating PKLR expression Molecular Cancer 18 1 145 10.1186/s12943-019-1078-z 31623628 PMC6796492

[b36-tjb-48-01-046] Zeng K He B Yang BB Xu T Chen X 2018 The pro-metastasis effect of circANKS1B in breast cancer Molecular Cancer 17 1 160 10.1186/s12943-018-0914-x 30454010 PMC6240936

[b37-tjb-48-01-046] Zhang M Han Y Zheng Y Zhang Y Zhao X 2020 ZEB1-activated LINC01123 accelerates the malignancy in lung adenocarcinoma through NOTCH signaling pathway Cell Death & Disease 11 11 981 10.1038/s41419-020-03166-6 33191397 PMC7667157

[b38-tjb-48-01-046] Zheng Q Bao C Guo W Li S Chen J 2016 Circular RNA profiling reveals an abundant circHIPK3 that regulates cell growth by sponging multiple miRNAs Nature Communications 7 11215 10.1038/ncomms11215 PMC4823868 27050392

